# Asymmetric facial edema with transdermal fentanyl: when listening to patients and caregivers helps to diagnose

**DOI:** 10.1017/S1478951524002177

**Published:** 2025-02-21

**Authors:** Carolina Simões, Miguel Julião, Patrícia Calaveiras, Paula Câmara, Eduardo Bruera

**Affiliations:** 1 Department of Palliative Medicine, Equipa Comunitária de Cuidados Paliativos, ULS Amadora/Sintra, Amadora, Portugal; 2 Department of Palliative Care, Rehabilitation, and Integrative Medicine, The University of Texas MD Anderson Cancer Center, Houston, TX, USA

**Keywords:** Facial edema, fentanyl, diagnosis, caregivers, palliative care

## Abstract

**Objectives:**

The link between opioids and peripheral edema has been discussed in the literature, though scarcely, especially in case reports involving patients using transdermal fentanyl for pain management.

**Methods:**

We present a case of a 51-year-old man with advanced head and neck cancer who developed severe, asymmetrical left-sided hemifacial edema following the initiation of transdermal fentanyl for pain management, which subsequently subsided after switching to transdermal buprenorphine.

**Results:**

We reduced the fentanyl patch from 75 to 62.5 mcg/h. At a follow-up visit within 48 hours there was some improvement in the swelling of the eyelids and tongue, but no significant change was noted in the lips, chin, and cheek region; and the patient experienced facial pain and discomfort due to swelling. It was then decided to rotate the opioid to buprenorphine transdermal patch 52.5mcg/h every 3 days; and a rapid improvement in the patient’s face, particularly in the eyelids and cheek region was observed. The remaining edema with the buprenorphine patch could be due to cancer progression.

**Significance of results:**

The final diagnosis of edema as a side effect of transdermal fentanyl was reached through careful knowledge of the frequent and non-frequent side effects of opioid drugs, clinical observation and, importantly, by listening to the patient and his wife, whose insights and observations were integrated with the medical team’s knowledge and assessments. Our report enhances the benefit of paying close attention to the input and observations of patients and caregivers, as they are the ones most familiar with the disease’s impact on daily life and the subtle changes and details that may go unnoticed in the clinical setting.^‡^

## Introduction

The link between opioids and peripheral edema especially with case reports involving patients using transdermal fentanyl for pain management has been discussed in the literature albeit scarcely (Gardner-Nix 2002; O’Conor et al. 1991; Veizi et al. 2016).

Given the understanding that opioids can contribute to peripheral edema, we present the case of a 51-year-old man with advanced head and neck cancer who developed severe, asymmetrical left-sided hemifacial edema following the initiation of transdermal fentanyl for pain management, which subsequently subsided after switching to transdermal buprenorphine.

The final diagnosis of edema as a side effect of transdermal fentanyl was reached through careful clinical observation and, importantly, by listening to the patient and his wife, whose insights and observations were integrated with the medical team’s knowledge and assessments. Our case report received ethical approval (Rapport 290/CEFMUP/2024).

## Case description

**Figure 1.**
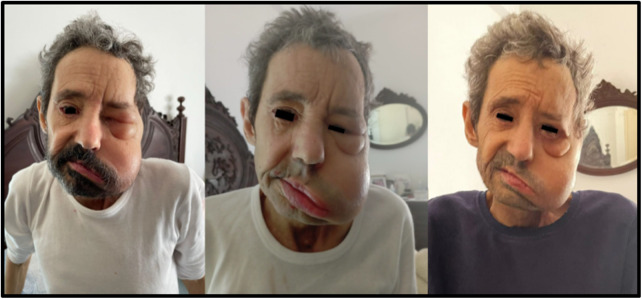
Left hemi-face edema during the use of transdermal fentanyl with severe eyelid, cheek region edema, and upper lip (left picture) and after opioid rotation to transdermal buprenorphine with significant reduction of eyelid, cheek region, chin, and lip edema (middle and right pictures).

A 51-year-old man who was referred to our home-based palliative care unit. He was suffering from severe sarcopenia and facial pain caused by left pyriform sinus carcinoma, as well as pain due to a deep sacral bedsore. Upon presentation, he was using a transdermal fentanyl patch 75 mcg/h every 72 hours, 25 mg of pregabalin tid (already as a chronic medication for anxiety and later as pain adjuvant), 12 mg senna bid as laxatives, clonazepam 0.5 mg at bedtime for insomnia, 0.5 mg for anxiety PRN q8h, and immediate release 20 mg/ml oral solution morphine for breakthrough pain q4h. Renal function, fluid balance and electrolytes were normal. At the first home visit, the team was alerted by the patient’s wife (and corroborated by the patient as well), that her husband, who already had minor facial swelling due to his left pyriform sinus carcinoma, experienced intensified facial swelling after being starting the 75 mcg/h fentanyl patch. Within about 8 hours of applying the patch in the hospital pain management consultation, his left hemi-face swelled intensely, causing watery and translucent edema, closing his eyelids, severely affecting his lips, chin, and cheek region, and also causing tongue edema (Figure 1). She noticed that this effect would gradually improve over the course of the 72-hour duration of the transdermal patch, with the swelling and pain decreasing significantly by the third day. However, the edema returned 6–8 hours after applying the new patch. The hemifacial and tongue swelling, pain was accompanied by sialorrhea.

The wife was convinced that the severe edema was due to the fentanyl patch initiation. She mentioned that she “knew her husband had some left-hemifacial edema due to cancer before, but it had not been as severe as it was now.” She explained that “she was with him, taking care of him, and both she and her daughter had noticed the change every time.”

The team became aware of the need to investigate the causality of the patient’s facial swelling after initiating the fentanyl patch. Using the Naranjo Adverse Drug Reaction Probability Scale, the score was 5, indicating probable causality. In order to conduct a therapeutic reduction trial without compromising the patient’s pain control, the fentanyl patch was reduced to 62.5 mcg/h after discussing it with the patient and their family. At a follow-up visit within 48 hours, there was some improvement in the swelling of the eyelids and tongue, but no significant change was noted in the lips, chin, and cheek region (Figure 1). The patient experienced facial pain and discomfort due to swelling. As a result, it was decided to rotate the opioid to buprenorphine transdermal patch 52.5 mcg/h every 3 days. There was rapid improvement in the patient’s face, particularly in the eyelids and cheek region. The patient’s wife informed the team that her husband’s face had almost returned to its state before the fentanyl treatment, although she was aware that the remaining edema with the buprenorphine patch could be due to cancer progression. After reducing the swelling of his face, particularly the edema, watery and fragile skin that made him afraid to cut and injure himself, the patient was able to shave his beard again and regain that sense of self-esteem. Informed proxy consent (wife) was obtained as the report was written after the terminally ill person’s death.

## Discussion

The wise words of Sir William Osler still hold true today: “Listen to your patient. He is telling you the diagnosis” (Osler 1920). We would also add, “Listen to the caregivers or loved ones of the patients. They are also telling you the diagnoses.” One easily ignores the family’s observation and attributes facial edema solely to cancer progression. Actively listening and discussing possible alternatives, making the wife part of the clinical team it became possible to improve this very uncomfortable symptom.

As far as we know, this is a rare case describing facial edema caused by transdermal fentanyl. The majority of published cases of peripheral edema caused by this drug are located in the lower and upper extremities (Conley et al. 2023; Gardner-Nix 2002; Radparvar 2022; Veizi et al. 2016). We hypothesize that our patient had severe half-face edema due to altered anatomy of his head and neck because of the pyriform carcinoma, causing impaired fluid reabsorption, and fluid accumulation due to altered vascular permeability. Another contributing factor to the asymmetric localization, in addition to the latter, was the patient’s habit of spending most of the day lying on his left side (to ease the pain of his infected and deep sacral bedsore), which aggravated dependent edema on the left side of his face. Although we think this was not a probable case, we can also speculate that adding an opioid to pregabalin might have contributed to the facial edema due to the opioid slowing the gastric transit and allowing more absorption of the pregabalin. As underlined by Gardener-Nix, managing fentanyl-induced peripheral edema should involve dose adjustment or switch to alternative pain medications. Our case demonstrates that opioid rotation was beneficial. The rapid improvement with another opioid such as buprenorphine suggests that fentanyl may cause edema by non-opioid effects. More research is needed to characterize the possible mechanisms for this side effect.

Gabapentinoids have been found to cause peripheral edema (Finegan et al. 2020; Gallagher and Apostle 2013; Read et al. 2021), and it is possible that the combination between pregabalin and fentanyl increased the asymmetric edema. However, there was rapid improvement after the opioid rotation, suggesting that pregabalin was not a major contributor to edema in this patient.

Considering the specific anatomical changes and altered vascular permeability caused by cancer is crucial and should prompt healthcare providers to be suspicious of potential asymmetric fentanyl-induced edema.

Our case report highlights the frequently overlooked and underdiagnosed side effect of peripheral edema associated with opioid therapy. This side effect may go unnoticed, especially if it occurs asymmetrically and is suspected to be closely related to specific anatomical changes due to the mass effect of cancer on surrounding tissues. Since opioids are commonly prescribed for the management of chronic pain, it is important for clinicians, patients, and caregivers to keep in mind edema as another possible side effect.

Clinical alertness regarding rare and asymmetrical side effects of opioid treatments should be taken attentively by providers. Frequent and close observation is essential, supported by tools such as the Naranjo Scale for addressing possible adverse drug reactions, drug dose reduction, and opioid rotation. But clinicians should also and always remember to pay close attention to the input and observations of patients and caregivers, as they are the ones most familiar with the disease’s impact on daily life and the subtle changes and details that may go unnoticed in clinical settings.

The mechanism of opioid-induced edema is unclear and multifaceted; therefore, future research is needed to broaden our understanding of opioid drugs and their possible side effects as they are primordial to control severe pain.

## Supporting information

Simões et al. supplementary material Simões et al. supplementary material

## Supplementary material

The supplementary material for this article can be found at https://doi.org/10.1017/S1478951524002177.
