# Asymmetric facial edema with transdermal fentanyl: when listening to patients and caregivers helps to diagnose – ERRATUM

**DOI:** 10.1017/S1478951525000379

**Published:** 2025-06-03

**Authors:** Carolina Simões, Miguel Julião, Patrícia Calaveiras, Paula Câmara, Eduardo Bruera

**Keywords:** Facial edema, fentanyl, diagnosis, caregivers, palliative care, erratum

The Publisher apologises for publishing this article with an incorrect Abstract. It has been corrected in the published paper, and it is also shown here:

**Abstract**

**Objectives.** The link between opioids and peripheral edema has been discussed in the literature, though scarcely, especially in case reports involving patients using transdermal fentanyl for pain management.

**Methods.** We present a case of a 51-year-old man with advanced head and neck cancer who developed severe, asymmetrical left-sided hemifacial edema following the initiation of transdermal fentanyl for pain management, which subsequently subsided after switching to transdermal buprenorphine.

**Results.** We reduced the fentanyl patch from 75 to 62.5 mcg/h. At a follow-up visit within 48 hours there was some improvement in the swelling of the eyelids and tongue, but no significant change was noted in the lips, chin, and cheek region; and the patient experienced facial pain and discomfort due to swelling. It was then decided to rotate the opioid to buprenorphine transdermal patch 52.5mcg/h every 3 days; and a rapid improvement in the patient’s face, particularly in the eyelids and cheek region was observed. The remaining edema with the buprenorphine patch could be due to cancer progression.

**Significance of results.** The final diagnosis of edema as a side effect of transdermal fentanyl was reached through careful knowledge of the frequent and non-frequent side effects of opioid drugs, clinical observation and, importantly, by listening to the patient and his wife, whose insights and observations were integrated with the medical team’s knowledge and assessments. Our report enhances the benefit of paying close attention to the input and observations of patients and caregivers, as they are the ones most familiar with the disease’s impact on daily life and the subtle changes and details that may go unnoticed in the clinical setting.

## References

[ref1] Simões C, Julião M, Calaveiras P, Câmara P and Bruera E (2025) Asymmetric facial edema with transdermal fentanyl: when listening to patients and caregivers helps to diagnose. *Palliative & Supportive Care*. Published by Cambridge University Press, 21 February 2025. doi:10.1017/S1478951524002177 PMC13166521 39980142

