# Supplementary material for: Asymmetric facial edema with transdermal fentanyl: when listening to patients and caregivers helps to diagnose
Source: Palliat Support Care. 2025 Feb 21;23:e67. doi: 10.1017/S1478951524002177 (PMC13166521; doi:10.1017/S1478951524002177)
Supplement: Simões et al. supplementary material [file S1478951524002177sup001.pdf]

Consentimento Informado para publicação de material identificável

Consentimento de Maria da Conceição dos Santos Correia, portadora do cartão de cidadão nº: 07480007, familiar de João Filipe Mateus Resende, falecido 23/05/2024, para a publicação de caso clínico e três fotografias

Nome da pessoa descrita no artigo ou mostrada na fotografia: João Filipe Mateus Resende
Objeto da fotografia(s) presente(s) no artigo: três fotografias seriadas e anexas a este consentimento informado, mostrando a evolução do inchaço da cara (metade esquerda da cara) antes e durante a utilização de fentanilo transdérmico e após a mudança para buprenorfina transdérmica.

Eu, Maria da Conceição dos Santos Correia, dou o meu consentimento para publicar a informação clínica anonimizada e as três fotografias do meu marido João Filipe Mateus Resende, presentes no artigo *Asymmetric facial edema with transdermal fentanyl* para aparecer na versão impressa e/ou eletrónica

Compreendo que o nome do meu marido não será publicado (nem serão publicadas as iniciais do seu nome), e que, mesmo assim, o completo anonimato pode não ser garantido, já que consta a sua idade e o seu diagnóstico principal para além das fotografias.

☒ Li todo o manuscrito e o que este contém e revi as fotografias, que serão publicadas e nas quais está o meu marido parente incluído.

Ou

☐ Foi-me dada a oportunidade de ler todo o manuscrito e ver todas as fotografias, nas quais está o meu marido incluído, e renunciei ao meu direito de o fazer.

☒ Fui informada que posso retirar a minha permissão a qualquer momento antes da publicação do artigo. Foi-me também explicado que, uma vez que o artigo tenha sido aceite para publicação, não será possível retirar a minha permissão.

☒ Foi-me fornecida uma folha de informação geral sobre a publicação.

☒ Confirmo que recebi uma cópia deste consentimento informado.

Assinatura da parente de João Filipe Mateus Resende: [signature]

Data: 31 / 7 / 2024

Grau de parentesco com João Filipe Mateus Resende: Esposa

Assinatura do Autor correspondente: [signature]

Nom 45734
